# On the association of common and rare genetic variation influencing body mass index: a combined SNP and CNV analysis

**DOI:** 10.1186/1471-2164-15-368

**Published:** 2014-05-14

**Authors:** Roseann E Peterson, Hermine H Maes, Peng Lin, John R Kramer, Victor M Hesselbrock, Lance O Bauer, John I Nurnberger, Howard J Edenberg, Danielle M Dick, Bradley T Webb

**Affiliations:** Virginia Institute for Psychiatric and Behavioral Genetics, Department of Human and Molecular Genetics, School of Medicine, Virginia Commonwealth University, Biotech I, 800 E. Leigh Street, Richmond, VA 23298-0126 USA; Virginia Institute for Psychiatric and Behavioral Genetics, Department of Human and Molecular Genetics, Massey Cancer Center, School of Medicine, Virginia Commonwealth University, Richmond, VA 23298 USA; Department of Psychiatry, Washington University, St. Louis, MO 63105 USA; Department of Psychiatry, University of Iowa, Iowa City, IA 52240 USA; Department of Psychiatry, School of Medicine, University of Connecticut, Farmington, CT 06030 USA; Institute of Psychiatric Research, Department of Psychiatry, School of Medicine, Indiana University, Indianapolis, IN 46226 USA; Department of Biochemistry and Molecular Biology, School of Medicine, Indiana University, Indianapolis, IN 46226 USA; Virginia Institute for Psychiatric and Behavioral Genetics, Virginia Commonwealth University, Richmond, VA 23298 USA; Virginia Institute for Psychiatric and Behavioral Genetics, Department of Psychiatry, School of Medicine, Virginia Commonwealth University, Richmond, VA 23298 USA

**Keywords:** Body mass index, Obesity, Genome-wide association, Copy number variation, Risk prediction, Polygenic score, *FTO*, *MC4R*

## Abstract

**Background:**

As the architecture of complex traits incorporates a widening spectrum of genetic variation, analyses integrating common and rare variation are needed. Body mass index (BMI) represents a model trait, since common variation shows robust association but accounts for a fraction of the heritability. A combined analysis of single nucleotide polymorphisms (SNP) and copy number variation (CNV) was performed using 1850 European and 498 African-Americans from the Study of Addiction: Genetics and Environment. Genetic risk sum scores (GRSS) were constructed using 32 BMI-validated SNPs and aggregate-risk methods were compared: count versus weighted and proxy versus imputation.

**Results:**

The weighted SNP-GRSS constructed from imputed probabilities of risk alleles performed best and was highly associated with BMI (p = 4.3×10^−16^) accounting for 3% of the phenotypic variance. In addition to BMI-validated SNPs, common and rare BMI/obesity-associated CNVs were identified from the literature. Of the 84 CNVs previously reported, only 21-kilobase deletions on 16p12.3 showed evidence for association with BMI (p = 0.003, frequency = 16.9%), with two CNVs nominally associated with class II obesity, 1p36.1 duplications (OR = 3.1, p = 0.009, frequency 1.2%) and 5q13.2 deletions (OR = 1.5, p = 0.048, frequency 7.7%). All other CNVs, individually and in aggregate, were not associated with BMI or obesity. The combined model, including covariates, SNP-GRSS, and 16p12.3 deletion accounted for 11.5% of phenotypic variance in BMI (3.2% from genetic effects). Models significantly predicted obesity classification with maximum discriminative ability for morbid-obesity (p = 3.15×10^−18^).

**Conclusion:**

Results show that incorporating validated effect sizes and allelic probabilities improve prediction algorithms. Although rare-CNVs did not account for significant phenotypic variation, results provide a framework for integrated analyses.

**Electronic supplementary material:**

The online version of this article (doi:10.1186/1471-2164-15-368) contains supplementary material, which is available to authorized users.

## Background

Obesity, defined clinically by a body mass index (BMI) ≥ 30 kg/m^2^, is a serious public health problem that occurs in over 1/3 of American adults [1, 2] and is associated with numerous medical conditions including cardiovascular disease [3], type II diabetes [4], and cancer [5]. Although nutritional intake and physical activity are known to affect relative body weight, twin and family studies have consistently shown a significant genetic contribution to body composition with heritability estimates of 40 to 70% [6].

Genome-wide association studies (GWAS) have successfully identified single nucleotide polymorphisms (SNPs) that contribute to individual variation in BMI and common obesity [7, 8]. In general adult populations of European descent, there are 32 SNPs showing robustly replicated association with BMI. However, individual variants have relatively small effects (0.06 to 0.39 kg/m^2^ in BMI per risk allele among Europeans) and in aggregate account for only a limited proportion of the phenotypic variance (~1.45%) [9]. GWAS of BMI in populations of African ancestry are limited but initial reports suggest a portion of the European-associated variants may also be associated across diverse populations [10–14].

Whereas reported single marker associations account for only a limited fraction of trait variance, linear mixed model approaches simultaneously consider the effects of common variation across the entire genome. As applied to BMI, this approach has demonstrated that common SNPs account for up to 17% of the phenotypic variance in BMI [15]. However, given that reported heritability estimates for BMI are typically much higher (40-70% [6]), a substantial proportion of the variance remains unaccounted for. To what extent this “missing heritability” is attributable to rare or structural variation is increasingly of interest to researchers and supported by a growing list of rare copy number variants (CNV) reported to be associated with BMI and obesity [16–24].

Given the widening spectrum of genetic variation demonstrated to be associated with common, complex traits, there is a need for genetic models integrating common and rare variants. In this study, we constructed a model that jointly incorporated the effects of common and rare (<1%) variants shown previously to be associated with obesity. First, genetic variants associated with BMI and obesity were catalogued from the literature, including common SNPs and common and rare CNVs. Next, genetic risk sum scores (GRSS), which summarize the total number of risk variants, were tested for association with BMI in 1850 Americans of European (EA) and 498 African (AA) descent from the Study of Addiction: Genetics and Environment (SAGE). Finally, we evaluated clinical utility of these models on the basis of discriminative ability to predict obesity classification.

## Methods

### Participants and phenotypes

Participants were from the Study of Addiction: Genes and Environment (SAGE) [25]. All SAGE participants provided written informed consent for genetic studies and agreed to share their DNA and phenotypic information for research purposes. All samples were de-identified and only subjects who consented to health research were included. The institutional review boards at all data collection sites granted approval for use of the data (Washington University in St. Louis, Henry Ford Health Sciences Center, Indiana University, The State University of New York Downstate Medical Center, University of Connecticut Health Center, University of California San Diego).

Study variables were assessed by interview, using versions of the Semi-Structured Assessment for the Genetics of Alcoholism (SSAGA) [26]. BMI was calculated from self-reported height and weight. Participants were removed from data analysis if they had missing data on either height or weight, height was < 1.4 or > 2 meters, weighed < 38 or > 166 kg, or if calculated BMI was < 14.5 or > 60 kg/m^2^, as values not in these ranges were likely due to data entry errors or suggestive of eating or syndromic disorders (n = 12). Clinical bodyweight categories were defined as overweight (BMI ≥ 25 kg/m^2^), obese class I (BMI ≥ 30 kg/m^2^), II (BMI ≥ 35 kg/m^2^) and III (BMI ≥ 40 kg/m^2^). Age was included as age at interview in years. Alcohol dependence (AD) was defined by the SSAGA according to DSM-IV criteria [27] and nicotine dependence (ND) was defined as having a Fagerström Test for Nicotine Dependence score of 4 or greater as assessed from the SSAGA.

Complete data on height, weight, AD, ND, genotypes and CNVs were available for 1850 EA and 498 AA participants. Descriptive statistics for study variables are presented by sex and self-reported ancestry in Table 1. There was a significant race by sex interaction with BMI (*t-test* = 6.84, p = 1.01×10^−11^) indicating that females and AAs tended to have greater BMI. Males were more likely to be AD (*χ*^2^ = 286.02, p = 3.65×10^−64^) and ND (*χ*^2^ = 9.36, p = 0.002). The age by AD interaction was also significant (*t-test* = −3.11, p = 0.002) indicating that older subjects were less likely to be AD.

**Table 1:** **Descriptive statistics by sex and self-reported ancestry**

Variable	Men		Women	
	***Mean EA (SD) n = 780***	***Mean AA (SD) n = 231***	***Mean EA (SD) n = 1070***	***Mean AA (SD) n = 267***
*Age*	40.4 (9.7)	41.1 (8.3)	39.3 (9.0)	39.3 (6.9)
*BMI*	27.5 (4.6)	28.4 (5.1)	26.5 (6.5)	31.5 (7.3)
	***n (%)***	***n (%)***	***n (%)***	***n (%)***
*Obese*	184 (23.6%)	72 (31.2%)	246 (23.0%)	130 (48.7%)
*AD*	501 (64.2%)	171 (74.0%)	300 (28.0%)	120 (44.9%)
*ND*	406 (52.1%)	125 (54.1%)	466 (43.6%)	151 (56.6%)

Note: EA = European-American, AA = African-American, SD = standard deviation, Age = age at interview in years, BMI = body mass index kg/m^2^, Obese = BMI ≥ 30 kg/m^2^, AD = alcohol dependence, ND = nicotine dependence.

#### Genotyping

Samples were genotyped on the Illumina Human 1 M beadchip at the Center for Inherited Diseases Research at Johns Hopkins University. Details of quality control procedures have been previously reported [25]. Analysis was restricted to SNPs with minor allele frequency ≥ 1%, call rate ≥ 98% and Hardy-Weinberg Equilibrium p-value ≥ 10^−5^. IMPUTE2 was used to phase the observed genotypes and impute unobserved genotypes [28, 29] using the 1000 Genomes phase 1 reference panel (release June 2011, b37) [30] separately by ancestry. To minimize effects of population stratification, 577,039 SNPs were used to generate ten principal components (PC) using EIGENSOFT 3.0 [31] and SMARTPCA [32]. To circumvent over-fitting only PCs that were associated with BMI and indicative of ancestral background were used in subsequent analyses [31–33]. The software Quanto was used to assess the power of the SAGE sample (n = 2,348) to detect known BMI/obesity genetic variants [34]. These calculations were computed using descriptive statistics reported in original papers, which included variant frequency, effect size, odds-ratio and percent variance accounted for.

#### CNV calling

The Illumina 1 M array has 1,072,820 probes (which includes 23,812 non-SNP “intensity-only” markers) that were used for CNV detection. Three widely-used programs were used for CNV calling: CNVPartition (Illumina StudioBead software), PennCNV [35], and QuantiSNP [36]. Genomic waves were adjusted for CNVs called by PennCNV and QuantiSNP [37]. Both PennCNV and QuantiSNP report a metric score for quality control purposes and CNV calls with a Log Bayes Factor less than ten were removed as well as poor quality samples based on quality control measures for CNV analysis as described in our previous work [38]. CNV calls from the three programs were compared and integrated using Combined CNV (CNVision.org) [39]. To increase the positive predicative rate [38], only CNVs that were called by at least two programs, as defined by *≥* 50% reciprocal overlap, were analyzed. Given that calls in centromeric, telomeric and immunoglobin regions are prone to harbor false positives, CNV calls in those regions were removed from analyses (33 regions, 13941 calls) [35, 40].

### Selection of BMI/obesity-associated genetic variation

BMI SNPs were catalogued from a BMI meta-analyses by *Speliotes* and colleagues [9]. The meta-analyses identified 32 SNPs reaching genome-wide significance (p < 5x10^−8^) (Additional file 1: Table S1). The SAGE sample was not included in the meta-analysis and represents and independent sample to test BMI loci. Fifteen SNPs did not appear on the genotyping array. Ungenotyped markers were ascertained by two approaches in order to compare methods: 1) imputation and 2) proxy SNPs. Imputed SNPs analyzed had allele frequency greater than 1% (Additional file 1: Table S1) and imputation quality greater than 0.8. The proxy method used the LD structure of the genome to identify highly correlated SNPs that appear on the array as substitutes for the unobserved SNPs. Proxy SNPs were identified using SNP Annotation and Proxy Search V2.1 [41] using the HapMap release 22 CEU reference panel except for rs11847697, which did not have a highly correlated SNP (r^2^ < 0.7) and was therefore not included in SNP-GRSSs. Proxy SNP information appears in Additional file 1: Table S1b. BMI and obesity associated CNVs were catalogued from research published between January 2008 and January 2012 via PubMed search (Additional file 2: Table S2). Case reports, typical of monogenic inheritance, were not included in the catalogue as the focus of the current study was on common complex obesity.

### BMI SNP genetic risk sum scores

Primarily two methods exist for constructing genetic scores: count and weighted methods. The count method is the sum of the number of risk alleles, whereas the weighted method incorporates the sum of the number of risk alleles each weighted by its odds-ratio or effect size. In this study, the weighted scores were constructed from regression coefficients reported by *Speliotes et al.*[9]. Count and weighted scores using the proxy method were calculated using the profile option in PLINK [42]. If SNP information was missing in an individual then the scoring routine imputed expected values based on sample allele frequency. Count and weighted scores using imputed genotypes were constructed using R version 2.13.1(script available upon request to R.E.P.) [43]. Furthermore, to extend existing GRSS methodology [44], count and weighted scores were constructed using probabilities of imputed risk alleles (*p*) by the equation below (Equation 1). Count scores were calculated with β = 1 and weighted scores with β = effect size of each risk allele (A) reported by *Speliotes et al.*[9] summed over the number of risk alleles in the score (*n*). To determine if there was significant effect size differences by GRSS methodology z-scores were computed in R using Equation 2 and p-values assigned based on the standard normal distribution.12

#### CNV association

In the SAGE sample, CNVs with a frequency ≥ 1% were considered common, those with a frequency < 1% rare. Common BMI/obesity-associated CNVs were tested individually as well as in aggregate by count scores. The limited number of rare CNV variants expected to be detected in the SAGE sample made statistical analysis of individual rare CNVs inappropriate [45, 46]. Therefore, rare BMI/obesity-associated CNVs were tested by aggregate count scores (CNV-GRSSs). Additionally, since rare CNV burden scores have been associated with obesity [16, 19], the genome-wide load of rare CNVs was also tested by the count method. CNVs previously reported to be associated with BMI/obesity were considered the same region in the SAGE sample if the CNV boundaries shared at least 40% overlap with the CNV boundaries reported in the literature. Furthermore, since there is evidence that the positive predictive rate is increased for large CNVs, which is likely due to the increased number of probes in larger variants, common and rare scores were also constructed from CNVs ≥ 100-kb to potentially reduce the number of false positive calls in the score [38].

#### Linear models

R [43] was used to fit linear and logistic regression models using established covariates for BMI including PCs associated with BMI and ancestry, sex and age. AD and ND were also included as covariates since the SAGE sample was selected for these traits. Predictors in linear models were included in a stepwise process and independent variables were centered to facilitate interpretation of effects. Interactions between covariates and predictors were tested and included in the final model if the p-value of the interaction was less than the Bonferroni corrected significance level of 0.002.

### Prediction of obesity

To test whether the combined model of common and rare variation had clinical utility for obesity risk prediction, we assessed diagnostic efficiency by calculating the area under the (AUC) receiver operator criteria (ROC) curves, which is a plot of the true positive rate (sensitivity) against the false positive rate (1 - specificity). Binary logistic regression was used to calculate predicted probabilities of the models. SPSS Statistics version 19.0 was used for AUC analyses and the StAR software was used to test for statistical differences between ROC curves [47].

## Results

### BMI SNP-GRSS

**Figure 1:**
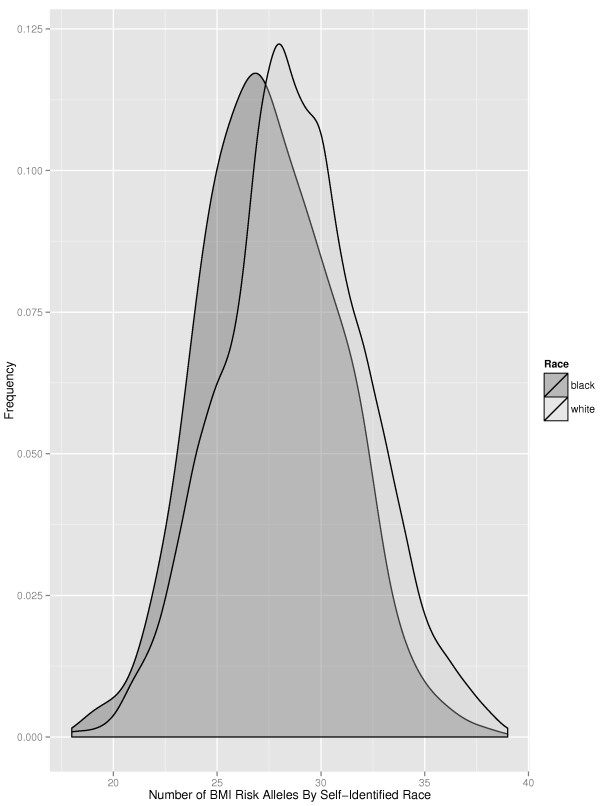
**Distribution of BMI-risk alleles by ancestry.** Note: BMI = body mass index kg/m^2^.

Seven of the 32 BMI-SNPs were found to be associated with BMI in the SAGE sample (p < 0.01), which included SNPs in or near *FTO* and *BDNF* (Additional file 1: Table S1). The mean number of BMI risk alleles per person was 28.5 (SD = 3.4) with a range from 18 to 39 and the distribution is presented by self-reported ancestry in Figure 1. As shown in Table 2, the SNP-GRSS was highly significantly associated with BMI in the combined sample (p < 1.11×10^−12^) and accounted for 3.1% of the variance. Examining GRSSs by ancestry indicated that point estimates for effect size and percent of variance accounted for in BMI tended to be greater in EA than AA sample (Additional file 3: Table S3a). However, there were no statistical differences in GRSS effect sizes (p > 0.138) when comparing by ancestry (Additional file 3: Table S3b). Although there were no statistical differences in effect sizes by GRSS method, the proportion of variance in BMI accounted for increased by 0.6-0.9% when using weighted scores and in the EA sample an additional 0.2% when incorporating imputed genotype probabilities (Additional file 3: Table S3c).

**Table 2:** **Comparison of the association of GRSSs with BMI constructed by count and weighted methods**

GRSS Method	***Mean (SD)***	***ES (SE)***	***T***	***p-value***	***R*** ^***2***^
*1. Proxy count*	0.450 (0.06)	0.927 (0.129)	7.18	9.07×10^−13^	0.022
*2. Proxy weighted*	0.063 (0.01)	1.104 (0.129)	8.56	2.05×10^−17^	0.027
*3. Imputed count*	0.447 (0.05)	0.865 (0.121)	7.16	1.11×10^−12^	0.022
*4. Imputed weighted*	0.062 (0.01)	1.035 (0.122)	8.51	2.94×10^−17^	0.030
*5. Imputed probability count*	0.894 (0.11)	0.872 (0.121)	7.21	7.33×10^−13^	0.022
*6. Imputed probability weighted*	0.124 (0.02)	1.037 (0.122)	8.54	2.43×10^−17^	0.031

Note: BMI = body mass index kg/m^2^, GRSS = genetic risk sum score, Mean = mean GRSS, ES = effect size for GRSS, Count = GRSS constructed from the summation of the number of risk alleles, Weighted = GRSS constructed from the number of risk alleles weighted by effect-sizes reported in *Speliotes* et al. 2010, SNP = single nucleotide polymorphism, Proxy = highly correlated substitute SNPs were used for variants not directly genotyped, Imputed = genotypes inferred from 1000 Genomes reference panel, Imputed probability = probability of genotypes inferred from 1000 Genomes reference panel.

#### CNV association

Eighty-four BMI/obesity-associated CNVs were catalogued from the literature and tested for association with BMI and obesity in the SAGE sample (Additional file 2: Table S2). Of the common CNVs, only a 21-kb deletion on 16p12.3 showed evidence for association with BMI (β = −0.057, p = 0.003, frequency = 16.9%). This CNV was also nominally associated with obese class I (OR = 0.743, p = 0.022) and II (OR = 0.630, p = 0.020). We would like to note that this CNV is correlated with SNP rs12444979, which was included in the GRSS (r = 0.798). However, since they were not in perfect LD and diagnostics between them did not suggest multicolinearity (variance inflation factor < 2.8) we chose to include both in subsequent analyses because it is possible that the SNP is capturing variation beyond the effect of the CNV. Additionally, rs2815752 near NEGR1 has been previously shown to tag a common deletion [9, 48, 49]. Although the SNP (included in the SNP-GRSS) was nominally associated (p =0.007) with BMI the CNV was not, which could be due in part to the low call rate of this deletion in SAGE (<1%). There were two additional common CNVs nominally associated with class II obesity. The first was a duplication on 1p36.1 (OR = 3.1, p = 0.009, frequency 1.2%) which ranged in length from 49.3 to 150.8 kb with a median value of 66.4 kb. The second was a large deletion on 5q13.2 (OR = 1.5, p = 0.048, frequency 7.7%) and ranged in length from 577.5 to 2238 kb with a median value of 1635 kb. None of the CNV-GRSSs, common or rare, were significantly associated with BMI or obesity in the SAGE sample. Descriptive statistics as well as association results for CNV-GRSSs are presented in Additional file 4: Table S4.

### Models incorporating effects of SNPs and CNVs

Results from linear regression analyses are displayed in Table 3. Model 1, which included the standard covariates, PC1 by sex and age by AD interactions but no genetic component, accounted for 8.3% of the variance in BMI. Model 2, which added the SNP-GRSS and the 21-kb deletion on 16p12.3 to the base model, fit significantly better [F_(3 2335)_ = 25.3, p = 3.34x^−54^] and accounted for an additional 3.2% of phenotypic variance (3.1% due to SNP-GRSS, 0.1% due to deletion on 16p12.3) in BMI for a total of 11.5%. Interactions between the covariates and the SNP-GRSS were not significant except for sex, which suggested that the SNP-GRSS was statistically similar in EA and AA and across age but tended to account for more of the variation in females. No significant interactions between the covariates and the 21-kb deletion on 16p12.3 were found, which indicated that the CNV was comparably associated with BMI in males and females, EA and AA and across the age range observed in SAGE. Additional file 5: Table S5 gives full model statistics by ancestry. We have also included in Additional file 5: Table S5d models with the two SNPs (rs12444979, rs2815752) that have been previously shown to tag CNVs removed from the SNP-GRSS and did not find any major differences in model fit (i.e.*;* [F_(12 2,335)_ = 25.34, p-value = 3.34×10^−54^, R^2^ = 0.115] vs. [F_(12 2,335)_ = 24.54, p-value = 1.97×10^−52^, R^2^ = 0.112]).

**Table 3:** **Linear models predicting BMI**

Model	Estimate EA	Estimate AA	Estimate combined	***p***-value EA	***p***-value AA	***p***-value combined
***Model 1: Covariates***						
Intercept	26.91	30.21	27.63	< 2×10^−16^	< 2×10^−16^	< 2×10^−16^
PC1	−50.11	−6.91	−98.82	0.788	0.939	2.40×10^−29^
PC4	19.31	−29.93	10.54	0.027	0.157	0.167
PC8	−3.18	−28.41	−30.20	0.934	0.008	0.002
Sex	−1.26	2.49	−0.46	1.76×10^−5^	3.67×10^−5^	0.081
Age	0.05	−0.01	0.04	2.13×10^−4^	0.984	9.45×10^−4^
AD	−0.15	−0.37	−0.20	0.062	0.018	0.004
ND	−0.10	0.07	−0.06	0.157	0.627	0.361
PC1*Sex	295.12	−249.00	−122.29	0.409	0.172	1.92×10^−12^
Age*AD	−0.02	−0.07	−0.02	0.026	0.0006	3.20×10^−4^
***Model 2: Covariates, GRSS & CNV***						
Intercept	26.91	30.22	27.63	< 2×10^−16^	< 2×10^−16^	< 2×10^−16^
PC1	−107.10	−14.09	−110.22	0.560	0.877	1.89×10^−35^
PC4	20.20	−30.04	10.14	0.019	0.153	0.176
PC8	11.44	−30.56	−31.53	0.765	0.004	8.36×10^−4^
Sex	−1.24	2.51	−0.43	1.70×10^−5^	2.89×10^−5^	0.099
Age	0.05	0.01	0.04	2.03×10^−4^	0.963	8.15×10^−4^
AD	−0.15	−0.37	−0.20	0.058	0.020	0.005
ND	−0.12	0.09	−0.07	0.087	0.566	0.253
PC1*Sex	170.80	−261.80	−131.38	0.627	0.150	3.91×10^−14^
Age*AD	−0.01	−0.06	−0.02	0.032	0.001	6.59×10^−4^
SNP-GRSS	65.40	42.30	62.44	2.55×10^−15^	0.036	4.30×10^−16^
Sex*SNP-GRSS	39.96	70.47	44.37	0.014	0.076	0.003
Del 16p12.3	−0.60	−0.61	−0.57	0.079	0.511	0.075

Note: BMI = body mass index kg/m^2^, Estimate = regression coefficient, EA = European-American, AA = African-American, GRSS = genetic risk sum score, PC = principal component score, Age = age at interview in years, AD = alcohol dependence, ND = nicotine dependence, CNV = copy number variation, Del = deletion.

* = interaction term.

### Obesity risk prediction

To test the discriminative accuracy of models to predict obesity classification, ROC curves were plotted and the corresponding AUCs were calculated. Three sets of nested models were tested: 1) covariates (PCs, sex, age, ancestry by sex interaction), 2) covariates, SNP-GRSS and interaction with sex and 3) covariates, SNP-GRSS and three obesity-associated CNVs (the 21 kb deletion on 16p.12.3, the 66 kb duplication on 1p36.1, and the 1440 kb deletion on 5q13.2). Table 4 displays fit statistics from ROC curve analysis by BMI category (Additional file 6: Table S6 displays by ancestry). AUC estimates indicated the models significantly predicted overweight and obesity classification with maximum discriminative ability when employing model 3 to predict class III obesity (AUC = 0.750, 95% CI = [0.702, 0.797]). Models that included genetic information had significantly greater AUCs than models only including covariates (Table 4).

**Table 4:** **Discriminative accuracy of covariates, SNP-GRSS and CNV predicting BMI category in European- and African-Americans**

Model	AUC	95% CI	Asy. Sig. of Model	Δ AUC	% Δ AUC	***p*** Δ AUC
***Overweight: n = 1443 (61.4%)***						
*1. Covariates*	0.679	[0.657,0.700]	2.68×10^−48^	-	-	-
*2. Model 1 + SNP-GRSS*	0.692	[0.671,0.714]	9.23×10^−56^	0.013	1.91%	*0.001*
*3. Model 2 + CNV*	0.694	[0.672,0.715]	1.27×10^−56^	0.002	0.28%	0.372
***Obese Class I: n = 632 (26.9%)***						
*1. Covariates*	0.621	[0.594,0.647]	2.74×10^−19^	-	-	-
*2. Model 1 + SNP-GRSS*	0.661	[0.637,0.686]	2.77×10^−33^	0.040	6.44%	*0.0001*
*3. Model 2 + CNV*	0.662	[0.638,0.687]	1.12x10^−33^	0.001	0.15%	0.662
***Obese Class II: n = 264 (11.2%)***						
*1. Covariates*	0.648	[0.610,0.685]	5.22×10^−15^	-	-	-
*2. Model 1 + SNP-GRSS*	0.681	[0.646,0.716]	6.97×10^−22^	0.033	5.09%	*0.025*
*3. Model 2 + CNV*	0.690	[0.656,0.725]	5.58×10^−24^	0.009	1.32%	0.123
***Obese Class III: n = 106, (4.5%)***						
*1. Covariates*	0.711	[0.660,0.762]	1.97×10^13^	-	-	-
*2. Model 1 + SNP-GRSS*	0.741	[0.692,0.790]	4.81×10^−17^	0.030	4.22%	*0.029*
*3. Model 2 + CNV*	0.750	[0.702,0.797]	3.15×10^−18^	0.009	1.21%	0.152

Note: BMI = body mass index kg/m^2^, SNP = single nucleotide polymorphism, SNP-GRSS = genetic risk sum score constructed from imputed probability of carrying 32 BMI-associated SNPs weighted by effect size reported in *Speliotes* et al. 2010, CNV = copy number variation, AUC = area-under the receiver operator criteria curve, Asy. Sig. = asymptotic significance, Δ AUC = change in AUC from previous model, % Δ AUC = percent change in AUC from previous model, *p* Δ AUC = statistical significance of change in AUC, Overweight = BMI ≥ 25 kg/m^2^, Obese I = BMI ≥ 30 kg/m^2^, Obese II = BMI ≥ 35 kg/m^2^, Obese III = BMI ≥ 40 kg/m^2^_,_ Covariates = PC1, PC4, PC8, sex, age, AD, ND, PC1*sex, age*AD, PC = principal component score, Age = age at interview, AD = alcohol dependence, ND = nicotine dependence.

## Discussion and conclusions

We have constructed an integrated model of common and rare variation catalogued from the literature and demonstrated its association with BMI in 1850 European-American and 498 African-American SAGE participants. This study is among the first to incorporate both SNPs and CNVs in a joint genetic analysis of BMI and obesity risk prediction. Our best- fitting model included standard covariates, SNP-GRSS and a 21-kb deletion on 16p12.3, and accounted for 11.5% of the phenotypic variance in BMI (p = 3.34×10^−54^).

The effects of 32 BMI-associated SNPs were incorporated via an aggregate risk score and accounted for up to 3.1% of the variance in BMI. Comparison of SNP-GRSS methodology indicated that a weighted score resulted in a 0.6-0.9% increase in the amount of variance accounted for. Furthermore, in the EA sample incorporating the probability of risk alleles from imputation further increased the amount of variance accounted for in BMI. The effect of the score tended to be lower in the AA sample. Due to the limited sample size of the AA group it could not be determined with confidence if indeed the effect of the score on BMI differed by ancestry. However, a study by *Belsky* et al. report that a genetic score of BMI-associated SNPs tended to be less significant in an AA sample compared to those from the EA sample [50]. These findings highlight the value of large-scale meta-analysis validation efforts to characterize effect sizes for genetic variants. Future research should test these methods for improved risk prediction in other complex traits and diseases and in diverse populations.

Of 84 BMI/obesity-associated CNVs catalogued from the literature, only 46 were detected in SAGE and only one, 16p12.3 deletion, was significantly associated with BMI. Speliotes et al. first reported the 16p12.3 deletion in a large-scale meta-analysis because a common BMI-decreasing allele was highly correlated with the same 21 kb deletion [9]. In the present study, the CNV was also moderately associated with obesity classes I and II. Additionally, two common CNVs on 1p36.1 and 5q13.2 were nominally associated with class II obesity. Our results did not yield additional support for the other BMI/obesity-associated CNVs, which might reflect limited power in the SAGE sample to detect the range of effect sizes, even when aggregate effects were considered. However, only 4 of the 84 CNVs identified from the literature have been associated with BMI/obesity in multiple studies. To that point, a recent study by Walters et al. attempted to replicate 18 BMI/obesity-associated CNVs and only replicated a rare 220 kb deletion on 16p11.2 [51]. Therefore, it is conceivable that the collections of CNVs examined here contained a greater number of false positives than true variants, thereby reducing the potential for replication by a risk score. Large-scale BMI/obesity-associated CNV meta-analyses are needed to validate reported variants and to accurately characterize the magnitude of their effects.

We also assessed whether the integrated models were clinically useful for obesity risk prediction. A model including standard covariates, SNP-GRSS and three obesity-associated CNVs demonstrated significant discriminative ability to predict overweight and obesity classification, with maximum discriminative ability when predicting class III obesity (AUC = 0.750). Other studies using SNP-GRSS to predict obesity have incorporated 8–32 SNPs and reported AUC estimates ranging from 0.574 to 0.597 [9, 50, 52–54]. Although our AUC estimates were statistically significant, they fell short of the threshold used in clinical practice for screening (0.8) and an important extension of this work is model validation in independent samples.

There are several possible extensions of the work presented here. First, SAGE participants consisted of a selected sample for substance-use behaviors. Although we have included AD and ND as covariates in all analyses, research has shown these phenotypes to have complex relationships with body composition [55, 56], and this may complicate interpretation. Future research should test for associations in both larger and population-based samples. An additional extension of this work is to incorporate variation detected from other obesity phenotypes such as waist-to-hip ratio [57, 58], extremes of the BMI trait distribution [59], and from diverse populations [14]. Additionally, fine mapping efforts are needed and will likely identify lower-frequency variants, which are typically not genotyped on commercial GWAS-arrays. Therefore a further extension of the work presented here is to include lower-frequency SNPs and INDELs identified by large-scale exome and genome sequencing efforts. Another important extension of an integrated model of BMI and obesity is to incorporate the moderating effects of the environment. At least two of the BMI-validated SNPs exhibit gene by environment interactions (GxE) [60, 61]. For example, a large meta-analysis found that in physically active adults the effect of the *FTO* risk allele on obesity was attenuated by 27% [62]. Given the considerable impact of the environment on body composition, future research needs to incorporate environmental variables into models of disease and risk prediction. Despite the potential limitations of the current study, this work provides a framework for integrating common and rare variation as both an alternative form of replication of genetic effects as well as for risk prediction of complex traits.

## Electronic supplementary material

Additional file 1: Table S1: Association results of 32 BMI SNPs. (XLS 76 KB)

Additional file 2: Table S2: CNVs catalogued from the literature and frequency in the SAGE sub-sample. (XLSX 27 KB)

Additional file 3: Table S3: Comparison of the association of GRSSs with BMI constructed by count and weighted methods by self-reported ancestry. (XLSX 38 KB)

Additional file 4: Table S4: Common and rare CNV-GRSS. (XLSX 14 KB)

Additional file 5: Table S5: Linear models predicting BMI by ancestry. (DOCX 104 KB)

Additional file 6: Table S6: Discriminative accuracy of covariates, SNP-GRSS and CNV predicting BMI category by self-reported ancestry. (DOCX 107 KB)
