# Supplementary material for: On the association of common and rare genetic variation influencing body mass index: a combined SNP and CNV analysis
Source: BMC Genomics. 2014 May 14;15(1):368. doi: 10.1186/1471-2164-15-368 (PMC4035084; doi:10.1186/1471-2164-15-368)
Supplement: Supplementary file 5 — Additional file 5: Table S5: Linear models predicting BMI by ancestry. (DOCX 104 KB) [file 12864_2013_6065_MOESM5_ESM.docx]

Table 5a: Linear models predicting BMI in the European-American sample

| **Model** | ***Estimate*** | | ***SE*** | ***T*** | ***p*-value** |
| --- | --- | --- | --- | --- | --- |
| ***Model 1: Covariates*** [F_(9 1,840)_ = 5.58, p-value = 1.26x10^-7^, R^2^ = 0.026] | | | | | |
| Intercept | 26.91 | | 0.13 | 200.88 | < 2x10^-16^ |
| PC1 | -50.11 | | 186.82 | -0.27 | 0.788 |
| PC4 | 19.31 | | 8.75 | 2.21 | 0.027 |
| PC8 | -3.18 | | 38.97 | -0.08 | 0.934 |
| Sex | -1.26 | | 0.29 | -4.30 | 1.76x10^-5^ |
| Age | 0.05 | | 0.01 | 3.71 | 2.13x10^-4^ |
| AD | -0.15 | | 0.08 | -1.87 | 0.062 |
| ND | -0.10 | | 0.07 | -1.42 | 0.157 |
| PC1*Sex | 295.12 | | 357.58 | 0.82 | 0.409 |
| Age*AD | -0.02 | | 0.01 | -2.22 | 0.026 |
| ***Model 2: Covariates, GRSS & CNV*** [F_(12 1,837)_ = 10.79, p-value < 2.2x10^-16^, R^2^ = 0.065] | | | | | |
| Intercept | | 26.91 | 0.13 | 204.90 | < 2x10^-16^ |
| PC1 | | -107.10 | 183.80 | -0.58 | 0.560 |
| PC4 | | 20.20 | 8.59 | 2.35 | 0.019 |
| PC8 | | 11.44 | 38.27 | 0.299 | 0.765 |
| Sex | | -1.24 | 0.29 | -4.31 | 1.70x10^-5^ |
| Age | | 0.05 | 0.01 | 3.72 | 2.03x10^-4^ |
| AD | | -0.15 | 0.08 | -1.89 | 0.058 |
| ND | | -0.12 | 0.07 | -1.71 | 0.087 |
| PC1*Sex | | 170.80 | 351.40 | 0.49 | 0.627 |
| Age*AD | | -0.01 | 0.01 | -2.14 | 0.032 |
| SNP-GRSS | | 65.40 | 8.20 | 7.98 | 2.55x10^-15^ |
| Sex*SNP-GRSS | | 39.96 | 16.31 | 2.45 | 0.014 |
| Del 16p12.3 | | -0.60 | 0.34 | -1.76 | 0.079 |

Note: BMI = body mass index kg/m^2^, GRSS = genetic risk sum score, PC = principal component score reflecting ancestral background, Age = age at interview, AD = alcohol dependence, ND = nicotine dependence, CNV = copy number variation, Del = deletion.

Supplemental Table 5b: Linear models predicting BMI in the African-American sample

| **Model** | ***Estimate*** | | ***SE*** | ***T*** | ***p*-value** |
| --- | --- | --- | --- | --- | --- |
| ***Model 1: Covariates*** [F_(9 488)_ = 6.54, p-value = 7.95x10^-9^, R^2^ = 0.107] | | | | | |
| Intercept | 30.21 | | 0.28 | 106.85 | < 2x10^-16^ |
| PC1 | -6.91 | | 90.9 | -0.07 | 0.939 |
| PC4 | -29.93 | | 21.12 | -1.42 | 0.157 |
| PC8 | -28.41 | | 10.71 | -2.65 | 0.008 |
| Sex | 2.49 | | 0.60 | 4.16 | 3.67x10^-5^ |
| Age | -0.01 | | 0.03 | -0.02 | 0.984 |
| AD | -0.37 | | 0.16 | -2.36 | 0.018 |
| ND | 0.07 | | 0.15 | 0.49 | 0.627 |
| PC1*Sex | -249.00 | | 182.00 | -1.37 | 0.172 |
| Age*AD | -0.07 | | 0.02 | -3.45 | 0.0006 |
| ***Model 2: Covariates, GRSS & CNV*** [F_(12 485)_ = 5.70, p-value = 3.23x10^-9^, R^2^ = 0.123] | | | | | |
| Intercept | | 30.22 | 0.28 | 107.46 | < 2x10^-16^ |
| PC1 | | -14.09 | 90.80 | -0.15 | 0.877 |
| PC4 | | -30.04 | 21.01 | -1.43 | 0.153 |
| PC8 | | -30.56 | 10.67 | -2.86 | 0.004 |
| Sex | | 2.51 | 0.60 | 4.22 | 2.89x10^-5^ |
| Age | | 0.01 | 0.04 | 0.05 | 0.963 |
| AD | | -0.37 | 0.16 | -2.34 | 0.020 |
| ND | | 0.09 | 0.15 | 0.57 | 0.566 |
| PC1*Sex | | -261.80 | 181.60 | -1.44 | 0.150 |
| Age*AD | | -0.06 | 0.01 | -3.27 | 0.001 |
| SNP-GRSS | | 42.30 | 20.15 | 2.10 | 0.036 |
| Sex*SNP-GRSS | | 70.47 | 39.75 | 1.77 | 0.076 |
| Del 16p12.3 | | -0.61 | 0.93 | -0.66 | 0.511 |

Note: BMI = body mass index kg/m^2^, GRSS = genetic risk sum score, PC = principal component score reflecting ancestral background, Age = age at interview, AD = alcohol dependence, ND = nicotine dependence, CNV = copy number variation, Del = deletion.

Table 5c: Linear models predicting BMI in the combined European-American and African-American samples

| **Model** | ***Estimate*** | | ***SE*** | ***T*** | ***p*-value** |
| --- | --- | --- | --- | --- | --- |
| ***Model 1: Covariates*** [F_(9 2,338)_ = 23.66, p-value = 4.58x10^-39^, R^2^ = 0.083] | | | | | |
| Intercept | 27.63 | | 0.12 | 227.36 | < 2x10^-16^ |
| PC1 | -98.82 | | 8.67 | -11.40 | 2.40x10^-29^ |
| PC4 | 10.54 | | 7.63 | 1.38 | 0.167 |
| PC8 | -30.20 | | 9.59 | -3.15 | 0.002 |
| Sex | -0.46 | | 0.26 | -1.75 | 0.081 |
| Age | 0.04 | | 0.01 | 3.31 | 9.45x10^-4^ |
| AD | -0.20 | | 0.07 | -2.81 | 0.004 |
| ND | -0.06 | | 0.06 | -0.91 | 0.361 |
| PC1*Sex | -122.29 | | 17.28 | -7.08 | 1.92x10^-12^ |
| Age*AD | -0.02 | | 0.01 | -3.60 | 3.20x10^-4^ |
| ***Model 2: Covariates, GRSS & CNV*** [F_(12 2,335)_ = 25.34, p-value = 3.34x10^-54^, R^2^ = 0.115] | | | | | |
| Intercept | | 27.63 | 0.12 | 231.26 | < 2x10^-16^ |
| PC1 | | -110.22 | 8.72 | -12.63 | 1.89x10^-35^ |
| PC4 | | 10.14 | 7.50 | 1.35 | 0.176 |
| PC8 | | -31.53 | 9.43 | -3.34 | 8.36x10^-4^ |
| Sex | | -0.43 | 0.26 | -1.65 | 0.099 |
| Age | | 0.04 | 0.01 | 3.35 | 8.15x10^-4^ |
| AD | | -0.20 | 0.07 | -2.81 | 0.005 |
| ND | | -0.07 | 0.06 | -1.14 | 0.253 |
| PC1*Sex | | -131.38 | 17.26 | -7.61 | 3.91x10^-14^ |
| Age*AD | | -0.02 | 0.01 | -3.41 | 6.59x10^-4^ |
| SNP-GRSS | | 62.44 | 7.62 | 8.19 | 4.30x10^-16^ |
| Sex*SNP-GRSS | | 44.37 | 15.19 | 2.92 | 0.003 |
| Del 16p12.3 | | -0.57 | 0.32 | -1.78 | 0.075 |

Note: BMI = body mass index kg/m^2^, GRSS = genetic risk sum score, PC = principal component score reflecting ancestral background, Age = age at interview, AD = alcohol dependence, ND = nicotine dependence, CNV = copy number variation, Del = deletion.

Table 5d: Linear models predicting BMI in the combined European-American and African-American samples (removed SNPs rs12444979 & rs2815752 which have been previously shown to tag CNV)

| **Model** | ***Estimate*** | | ***SE*** | ***T*** | ***p*-value** |
| --- | --- | --- | --- | --- | --- |
| ***Model: Covariates, GRSS (30 SNPs) & CNV*** [F_(12 2,335)_ = 24.54, p-value = 1.97x10^-52^, R^2^ = 0.112] | | | | | |
| Intercept | | 27.63 | 0.12 | 230.84 | < 2x10^-16^ |
| PC1 | | -108.74 | 8.72 | -12.47 | 1.31x10^-34^ |
| PC4 | | 9.70 | 7.50 | 1.29 | 0.197 |
| PC8 | | -31.02 | 9.45 | -3.28 | 0.001 |
| Sex | | -0.43 | 0.26 | -1.65 | 0.098 |
| Age | | 0.04 | 0.01 | 3.35 | 8.08x10^-4^ |
| AD | | -0.20 | 0.07 | -2.82 | 0.005 |
| ND | | -0.08 | 0.07 | -1.14 | 0.252 |
| PC1*Sex | | -130.30 | 17.29 | -7.54 | 6.89x10^-14^ |
| Age*AD | | -0.02 | 0.01 | -3.43 | 6.13x10^-4^ |
| SNP-GRSS | | 56.25 | 7.22 | 7.80 | 9.61x10^-15^ |
| Sex*SNP-GRSS | | 36.76 | 14.50 | 2.54 | 0.011 |
| Del 16p12.3 | | -0.91 | 0.32 | -2.83 | 0.005 |

Note: BMI = body mass index kg/m^2^, GRSS = genetic risk sum score comprised of 30 SNPs (removed rs12444979 & rs2815752), PC = principal component score reflecting ancestral background, Age = age at interview, AD = alcohol dependence, ND = nicotine dependence, CNV = copy number variation, Del = deletion.
