# Supplementary material for: On the association of common and rare genetic variation influencing body mass index: a combined SNP and CNV analysis
Source: BMC Genomics. 2014 May 14;15(1):368. doi: 10.1186/1471-2164-15-368 (PMC4035084; doi:10.1186/1471-2164-15-368)
Supplement: Supplementary file 6 — Additional file 6: Table S6: Discriminative accuracy of covariates, SNP-GRSS and CNV predicting BMI category by self-reported ancestry. (DOCX 107 KB) [file 12864_2013_6065_MOESM6_ESM.docx]

Supplemental Table 6: Discriminative accuracy of covariates, SNP-GRSS and CNV predicting BMI category by self-reported ancestry

| **BMI**  **Category** | **Model** | **Sample** | **n (%)** | **AUC** | **95% CI** | **Asy. Sig.** |
| --- | --- | --- | --- | --- | --- | --- |
| ***Overweight*** | 1. Covariates | All | 1442 (61.4%) | 0.679 | [0.657,0.700] | 2.68x10^-48^ |
|  |  | EA | 1045 (56.5%) | 0.641 | [0.616,0.666] | 2.36x10^-25^ |
|  |  | AA | 397 (79.7%) | 0.632 | [0.572,0.692] | 4.39x10^-5^ |
|  | 2. SNP-GRSS | All |  | 0.692 | [0.671,0.714] | 9.23x10^-56^ |
|  |  | EA |  | 0.661 | [0.636,0.686] | 1.69x10^-32^ |
|  |  | AA |  | 0.642 | [0.583,0.700] | 1.08x10^-5^ |
|  | 3. CNV | All |  | 0.694 | [0.672,0.715] | 1.27x10^-56^ |
|  |  | EA |  | 0.662 | [0.637,0.686] | 7.76x10^-33^ |
|  |  | AA |  | 0.643 | [0.583,0.700] | 8.99x10^-6^ |
| ***Obese I*** | 1. Covariates | All | 632 (26.9%) | 0.621 | [0.594,0.647] | 2.74x10^-19^ |
|  |  | EA | 430 (23.2%) | 0.569 | [0.537,0.600] | 1.60x10^-5^ |
|  |  | AA | 202 (40.6%) | 0.631 | [0.582,0.681] | 6.43x10^-7^ |
|  | 2. SNP-GRSS | All |  | 0.661 | [0.637,0.686] | 2.77x10^-33^ |
|  |  | EA |  | 0.632 | [0.602,0.662] | 1.14x10^-16^ |
|  |  | AA |  | 0.631 | [0.582,0.681] | 6.22x10^-7^ |
|  | 3. CNV | All |  | 0.662 | [0.638,0.687] | 1.12x10^-33^ |
|  |  | EA |  | 0.633 | [0.603,0.663] | 6.73x10^-17^ |
|  |  | AA |  | 0.638 | [0.589,0.688] | 1.58x10^-7^ |
| ***Obese II*** | 1. Covariates | All | 264 (11.2%) | 0.648 | [0.610,0.685] | 5.22x10^-15^ |
|  |  | EA | 164 (8.9%) | 0.573 | [0.527,0.619] | 0.002 |
|  |  | AA | 100 (20.1%) | 0.662 | [0.605,0.719] | 5.47x10^-7^ |
|  | 2. SNP-GRSS | All |  | 0.681 | [0.646,0.716] | 6.97x10^-22^ |
|  |  | EA |  | 0.631 | [0.586,0.675] | 3.24x10^-8^ |
|  |  | AA |  | 0.681 | [0.621,0.740] | 2.22x10^-8^ |
|  | 3. CNV | All |  | 0.690 | [0.656,0.725] | 5.58x10^-24^ |
|  |  | EA |  | 0.645 | [0.601,0.690] | 8.15x10^-10^ |
|  |  | AA |  | 0.697 | [0.638,0.757] | 1.04x10^-9^ |
| ***Obese III*** | 1. Covariates | All | 106 (4.5%) | 0.711 | [0.660,0.762] | 1.97x10^13^ |
|  |  | EA | 60 (3.2%) | 0.650 | [0.584,0.716] | 7.36x10^-5^ |
|  |  | AA | 46 (9.2%) | 0.697 | [0.616,0.777] | 1.11x10^-5^ |
|  | 2. SNP-GRSS | All |  | 0.741 | [0.692,0.790] | 4.81x10^-17^ |
|  |  | EA |  | 0.711 | [0.643,0.779] | 2.51x10^-8^ |
|  |  | AA |  | 0.703 | [0.619,0.788] | 5.36x10^-6^ |
|  | 3. CNV | All |  | 0.750 | [0.702,0.797] | 3.15x10^-18^ |
|  |  | EA |  | 0.722 | [0.658,0.787] | 4.33x10^-9^ |
|  |  | AA |  | 0.712 | [0.632,0.791] | 2.25x10^-6^ |

Note: BMI = body mass index kg/m^2^, SNP = single nucleotide polymorphism, SNP-GRSS = genetic risk sum score constructed from imputed probability of carrying 32 BMI-associated SNPs by the weighted method, CNV = copy number variation, AUC = area-under the receiver operator criteria curve, Asy. Sig. = asymptotic significance, Overweight = BMI > 25 kg/m^2^, Obese I = BMI > 30 kg/m^2^, Obese II = BMI > 35 kg/m^2^, Obese III = BMI > 40 kg/m^2^_,_ Covariates = PC1, PC4, PC8, sex, age, AD, ND, PC1*sex, age*AD, PC = principal component score reflecting ancestral background, Age = age at interview, AD = alcohol dependence, ND = nicotine dependence.
